# Leveraging massively parallel reporter assays for evolutionary questions

**DOI:** 10.1186/s13059-023-02856-6

**Published:** 2023-02-14

**Authors:** Irene Gallego Romero, Amanda J. Lea

**Affiliations:** 1 Melbourne Integrative Genomics, University of Melbourne, Royal Parade, Parkville, Victoria 3010 Australia; 2 School of BioSciences, The University of Melbourne, Royal Parade, Parkville, 3010 Australia; 3 The Centre for Stem Cell Systems, Faculty of Medicine, Dentistry and Health Sciences, The University of Melbourne, 30 Royal Parade, Parkville, Victoria 3010 Australia; 4 Center for Genomics, Evolution and Medicine, Institute of Genomics, University of Tartu, Riia 23b, 51010 Tartu, Estonia; 5 Department of Biological Sciences, Vanderbilt University, Nashville, TN 37240 USA; 6 Vanderbilt Genetics Institute, Vanderbilt University, Nashville, TN 37240 USA; 7 Evolutionary Studies Initiative, Vanderbilt University, Nashville, TN 37240 USA; 8 Child and Brain Development Program, Canadian Institute for Advanced Study, Toronto, Canada

## Abstract

A long-standing goal of evolutionary biology is to decode how gene regulation contributes to organismal diversity. Doing so is challenging because it is hard to predict function from non-coding sequence and to perform molecular research with non-model taxa. Massively parallel reporter assays (MPRAs) enable the testing of thousands to millions of sequences for regulatory activity simultaneously. Here, we discuss the execution, advantages, and limitations of MPRAs, with a focus on evolutionary questions. We propose solutions for extending MPRAs to rare taxa and those with limited genomic resources, and we underscore MPRA’s broad potential for driving genome-scale, functional studies across organisms.

**Supplementary Information:**

The online version contains supplementary material available at 10.1186/s13059-023-02856-6.

## Introduction

A major goal in evolutionary biology is to understand why and how adaptively relevant traits differ between individuals and species. Recent advances in genomics have allowed researchers to make rapid progress in this area. In particular, advances in functional genomics have now clarified that changes in gene regulation are important for generating phenotypic variation both within and between species, and these changes frequently contribute to adaptation, speciation, and complex trait evolution [1–6]. Variation in gene regulation also underlies many fundamental biological processes, such as development, tissue differentiation, and the cellular response to environmental stimuli [7–9]. Consequently, there is great interest in harnessing emerging genomic technologies to address the role of gene regulation in evolutionary processes.

Gene regulatory programs are commonly orchestrated by *cis*-acting regulatory elements such as promoters, insulators, silencers, and enhancers (referred to from here on as “regulatory elements”). These elements are typically short sequences, on the order of 100s to 1000s of base pairs, that can be located within, close to, or distal to the genes they regulate (although in mammals they are often within 1 megabase [7, 10]). Enhancers and silencers in particular are defined by their ability to influence gene regulation regardless of their orientation to their target gene. Across all types of regulatory elements, transcriptional modulation is typically achieved by recruiting transcription factors and/or RNA polymerase II (e.g., this is a common function of promoter sequences) [7].

Regulatory elements in the human genome outnumber protein-coding genes by an order of magnitude [7] and allow for the induction of diverse and tissue- or context-specific transcriptional programs [8]. For example, upon infection, human monocytes upregulate NF-κB/Rel family transcription factors (TFs), which bind regulatory elements near innate immune genes resulting in mobilization of the cell’s defense program [11, 12]. Given the context-specific nature of a regulatory element’s function, mutations in these regions typically have fewer pleiotropic consequences relative to mutations in protein-coding genes, leading some to argue that they may be a preferred substrate of adaptive evolution [13, 14]. Indeed, regulatory elements have been shown to be evolutionarily important for generating morphological novelty in plants and animals [15, 16], for maintaining species barriers [5], and for establishing human-specific traits [17–19].

Despite the established significance of regulatory elements, studying them genome-wide has been difficult, especially outside of humans and model organisms. Any given element is likely to be active in a tissue or cell-type-specific manner, and tends to also exhibit context specificity (e.g., becoming active only at specific developmental stages or in response to a given external stimulus). In addition, regulatory elements are difficult to identify from genomic or epigenomic datasets: for example, enhancers display some predictable sequence features [20, 21] and associations with epigenetic marks (e.g., in humans and other vertebrates, they tend to be located in open chromatin regions, hypomethylated, and marked by H3K27ac and/or H3K4me1), but these features are not sufficient to predict enhancer activity nor are they exclusive to active enhancers [22, 23].

Thus, to confirm the identity, function, and strength of a putative regulatory element, experimental validation is required. Such tests commonly involve a “reporter assay”, in which a candidate sequence is cloned into a plasmid containing a minimal promoter and a reporter gene (e.g., GFP, LacZ, or luciferase). The plasmid is then transfected into a cell type of interest, where, if the candidate sequence is indeed a regulatory element, it will interact with the minimal promoter and result in differential expression of the reporter gene relative to a control construct that only contains the minimal promoter. Such approaches have provided important insight into candidate regulatory elements of evolutionary significance [24–26]. For example, Kvon and colleagues used a reporter assay to confirm that snake-specific mutations within the ZRS limb enhancer lead to a reduction in regulatory activity associated with limb loss [24]. While powerful, candidate sequences in this framework are unavoidably tested one by one, making the method laborious and impractical when there are many regions of interest, or when the discovery of genome-wide patterns is the goal. Recently developed methods, collectively known as “massively parallel reporter assays” (MPRAs), help fill this gap by enabling reporter assay experiments to be carried out in very high-throughput (e.g., testing thousands, hundreds of thousands, or millions of fragments simultaneously). However, due to technical and expertise-related hurdles, MPRAs have thus far been applied mainly to biomedical rather than evolutionary questions. They have also been restricted to a small number of species—namely humans and a few model organisms (e.g., fruit flies [27, 28] and mice [29]).

Our goal in this review is to showcase how MPRAs can be harnessed to improve our understanding of the generation and evolution of phenotypic diversity across the tree of life. To do so, we first provide an overview of MPRA data generation and analysis, as well as current applications of the approach; during this overview, we highlight the handful of existing studies that have harnessed MPRA technology for evolutionary questions. We then move to a discussion of study designs that could be leveraged to further address evolutionary questions. We also consider anticipated challenges and potential solutions for expanding MPRA protocols to non-model organisms. We tailor these discussions and recommendations specifically to evolutionary studies, with the aim of highlighting the payoffs of integrating MPRAs into this field.

## Overview of MPRA technologies

MPRAs grew out of saturation mutagenesis [30, 31] and *cis*-regulatory element screens [32, 33], which were developed to explore the effects of all possible point mutations in a candidate regulatory region. To do so, these protocols linked each of several thousand mutated sequences to a number of unique barcodes, with each sequence-barcode pair represented in a different reporter assay vector. After delivery of the pooled vector library into a cell type of interest, barcode abundance could be subsequently quantified through RNA-seq (and normalized to DNA-seq-based quantification of the input oligonucleotide pool). Together, this approach allows hundreds of different sequences to be tested simultaneously in a single experiment. For example, Patwardhan and colleagues explored the functional impact of every possible mutation in three mouse liver enhancers and found that activity was generally robust to sequence variation: only ~3% of mutations altered regulatory activity by more than two-fold [31]. The protocol innovations that enabled saturation mutagenesis of candidate regulatory elements (as performed by Patwardhan and colleagues) were quickly applied and optimized to create MPRAs—higher-throughput approaches that could not only test mutagenized sequences of candidate regulatory elements, but also naturally occurring polymorphisms at a genome-wide scale.

**Fig. 1:**
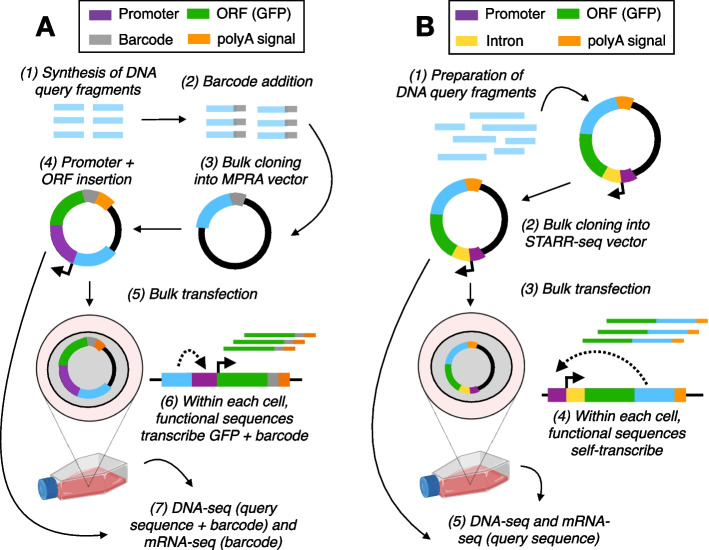
Overview of MPRA workflows. **A** (1) In the barcoded MPRA design, candidate regions of interest are synthesized via large-scale oligosynthesis. (2) The single-stranded DNA is paired with a unique barcode and converted to double-stranded DNA via PCR. (3) The barcoded DNA fragments are then cloned into an empty MPRA reporter vector. Next, the plasmid library is linearized between the barcode and the candidate query sequence, and (4) a minimal promoter (often SCP1) and open reading frame are inserted. (5) This plasmid pool is delivered via transfection (or infection if viral delivery is used) into the desired cell type, where (6) functional regulatory elements sequences will interact with the promoter to drive transcription of the ORF and the barcode, which is incorporated into each transcript’s 3′UTR. Finally, RNA is harvested from the cells, and (7) mRNA is sequenced to measure post-experiment barcode abundance, along with DNA fragments from the empty MPRA reporter vector step to identify query sequence-barcode associations. **B** (1) In the classic STARR-seq design, sequencing adapters as well as sequences complementary to the STARR-seq vector are added to DNA fragments of interest. (2) This fragment pool is then cloned into the STARR-seq vector upstream of a 3′ poly-adenylation signal and downstream of a promoter and synthetic intron (to differentiate spliced mSTARR-seq RNA transcripts from plasmid DNA in downstream PCRs). (3) After delivery into a cell line, (4) inserts that possess regulatory activity interact with the promoter to drive expression of the insert itself. Finally, RNA is harvested from the cells, and (5) mRNA is sequenced to measure post-experiment fragment abundance, along with DNA fragments from the pre-transfection (or pre-infection) plasmid pool to control for variation in input. See Fig. 2 for further information on data analysis

**Fig. 2:**
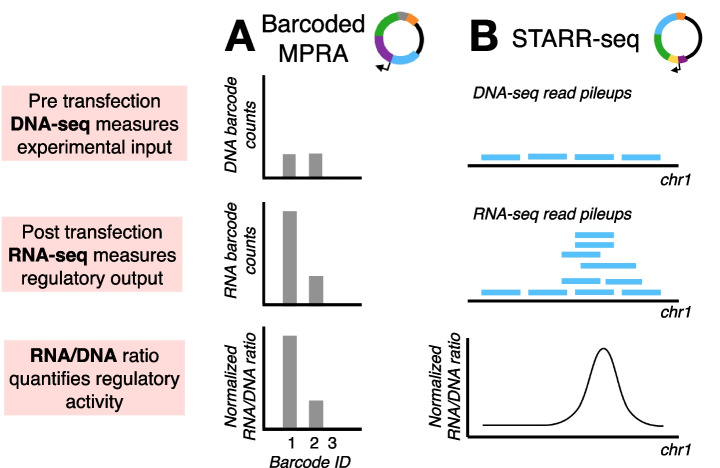
Overview of MPRA data and analysis. For both **A** barcoded MPRA and **B** STARR-seq experiments, pre-transfection (or pre-infection) plasmid-derived DNA is sequenced to control for variation in the pool of fragments input into the experiment, while post-experient plasmid-derived RNA is sequenced to measure regulatory output. In both cases, the post-experiment mRNA-to-DNA ratio therefore reflects regulatory activity controlling for variation in input. In **A** barcoded MPRAs, the sequencing target is the fragment-associated barcode, while in **B** STARR-seq experiments, the sequencing target is the query fragment itself

MPRAs consist of three main steps. First, DNA sequences of interest are synthesized and cloned in conjunction with a unique barcode into a specially engineered plasmid that contains a minimal promoter and a reporter gene. If barcodes are added during the cloning step, the library is then sequenced at high depth to associate barcodes to each assayed sequence. Second, the reporter library is transfected, or infected if delivered virally, into a cell type of interest; within each cell, plasmids containing active regulatory elements will transcribe the reporter gene and associated barcode. Finally, RNA is extracted from the pool of transfected cells, and high-throughput sequencing is used to quantify the barcoded region. In this design, RNA barcode abundance, after controlling for DNA input, thus scales quantitatively with the regulatory activity of a given tested sequence (Figs. 1 A and 2A).

A variation on this design is “self-transcribing active regulatory region sequencing” (STARR-seq), in which the sequence of interest is cloned into the plasmid downstream of a minimal promoter and reporter gene and upstream of a poly-A tail. Consequently, within a given cell, sequences with regulatory activity will interact with the promoter to drive expression of the reporter gene and the sequence itself. RNA abundance of the focal sequence, again after controlling for DNA input, thus reflects regulatory element strength (Figs. 1 B and 2B). This approach is similar to the MPRA design described above and in Fig. 1A, hereafter referred to as “barcoded MPRA”; however, STARR-seq circumvents the need for both barcodes and fragment synthesis (we note that both barcoded MPRA variations *without* fragment synthesis, and STARR-seq using synthetic constructs, are possible [34, 35], but uncommon).

Because fragment synthesis is not required, STARR-seq is typically more time- and cost-efficient than barcoded MPRAs for testing large libraries, such as those including randomly sheared as well as captured, immunoprecipitated, or otherwise selected genomic DNA fragments (Table 1). Nevertheless, barcoded MPRAs still have their advantages. For example, sequence-specific biases in mRNA stability can be a problem for inference via STARR-seq [48]; however, because barcoded MPRAs test each candidate regulatory element in association with multiple barcodes and with multiple tiled sequences over the element, this issue is much less of a concern.

**Table 1:** An overview of different MPRA approaches

Assay	Summary
*“Classic” methods*	
Barcoded MPRA [23, 31, 33, 36]	DNA sequences of interest are each synthesized in conjunction with a unique barcode and cloned into a plasmid upstream of a promoter, reporter gene, the unique barcode, and a poly-A tail. Sequences with regulatory activity drive expression of transcripts that include the barcode, such that barcode abundance in RNA extracted from transfected cells reflects regulatory element strength.
STARR-seq [27]	Sequences of interest are cloned into a plasmid downstream of a minimal promoter (or, more recently, simply the origin of replication) and reporter gene and upstream of a poly-A tail. Sequences with regulatory activity drive expression of transcripts that include the sequence itself, such that the abundance of the focal sequence in RNA extracted from transfected cells reflects regulatory element strength.
*Elaborations on the classic, barcoded MPRA design*	
Lenti-MPRA [37]	Lentivirus is used to integrate MPRA libraries into the genome, thereby circumventing concerns that episomal reporter assays carried out via transient transfection may not reflect gene regulatory processes that take place in a native chromatin context. The cell-type range of lentivirus transduction is also much broader than transient transfection, opening the door to experiments in hard-to-transfect cell types.
AAV MPRA [35]	MPRA libraries are packaged into an adeno-associated virus (AAV) for transfection. AAV is a nonpathogenic virus commonly used for gene therapy studies and permits transfection into a wide range of tissues, including post-mitotic tissues and tissues that are hard to transfect with traditional chemical or electrical methods. Unlike DNA delivered by lentivirus, the AAV-delivered DNA remains almost exclusively episomal.
Saturation mutagenesis-based MPRA [38]	To test the functional effects of thousands of mutations in a candidate regulatory element, error-prone PCR is used to introduce sequence variation and to incorporate random sequence tags. These constructs are then assayed via the MPRA design to pinpoint SNPs that affect regulatory activity.
*Elaborations on the classic STARR-seq design*	
STAP-seq [39]	Rather than measuring the activity of many candidate regulatory elements in the presence of a given minimal promoter, STAP-seq measures the responsiveness of many candidate promoters in the presence of a given element. Promoter candidates are cloned downstream of a strong enhancer and upstream of an ORF and poly-A tail. If a candidate fragment is capable of initiating transcription, it will produce reporter transcripts that start with the promoter candidate sequence wherever the TSS was initiated.
UMI-STARR-seq [40]	This protocol introduces unique molecular identifiers (UMI) prior to post-transfection amplification of cell-extracted mRNA. The UMIs allow the researcher to account for PCR duplicates in downstream analyses, and are recommended especially for low complexity input libraries.
ChIP-STARR-seq [41]	Open chromatin regions are incorporated into a DNA library, which is then assayed via STARR-seq.
Pop-STARR-seq [42]	Regions of interest are amplified from DNA derived from many unique individuals. These genetically diverse products are then pooled and used as the input for STARR-seq.
ATAC-STARR-seq [43]	Open chromatin regions are incorporated into a DNA library via ATAC-seq [44], and these elements are then assayed via STARR-seq. This design allows the researcher to preferentially test the activity of putative regulatory elements found within open chromatin in a given cell type.
BiT-STARR-seq [45]	Oligos covering each of the alleles for a set of SNPs of interest are synthesized and incorporated into STARR-seq experiments to test for allele-specific expression. UMIs are also added during cDNA synthesis to account for PCR duplicates.
mSTARR-seq [46]	STARR-seq style plasmid pools are constructed using a CpG-free reporter vector that retains the same functionality. Enzyme treatment is then used to create methylated and unmethylated versions of the plasmid pool, which can be assayed to identify regulatory sequences as well as methylation-dependent regulatory sequences.
CapSTARR-seq [47]	Putative regulatory elements are selected from genomic DNA using hybridization capture-based target enrichment. Captured regions are then assayed via STARR-seq, allowing the researcher to test a targeted set of fragments without relying on oligo synthesis.

Many variations on the barcoded (Fig. 1A) and STARR-seq flavor (Fig. 1B) of MPRA designs have been utilized in recent years, with protocol modifications focused on different ways to select DNA input for STARR-seq (e.g., ATAC-STARR-seq [43], ChIP-STARR-seq [41], CapSTARR-seq [47]), integrating MPRA plasmids into the endogenous genome (lentiMPRA [37]), incorporating methyl mark manipulations to test the effects of DNA methylation on regulatory function (mSTARR-seq [46]), or modifying the MPRA framework to study mRNA stability and alternative splicing [49–52]. These changes to the design impact the types of information that can be gained from a given assay (see Table 1 and ref [53] for a detailed comparison). Additionally, we note that in parallel to the developments we discuss in this review, recent years have seen the establishment of deep mutational scans, which test for the effects of all possible mutations in a coding sequence on protein function [54]. In some areas of the literature, MPRAs (both the barcoded and STARR-seq versions) and deep mutational scans have been grouped under the broader header of “multiplexed assays for variant effect” (MAVEs) [55, 56]. However, here we focus specifically on assays that consider gene regulation rather than protein function as the output, and we therefore use MPRA to describe the family of assays laid out in Table 1 and Fig. 1, rather than MAVE (see Additional file 1: Fig S1 for a terminology hierarchy).

## Overview of MPRA analyses

Once generated, analysis of MPRA data relies on diverse computational and statistical approaches, which we briefly overview here to familiarize the reader with the breadth of possible inferences. In both the barcoded MPRA and STARR-seq designs, RNA sequencing is performed to assess the transcription rate of each query fragment, while DNA sequencing is performed to assess the diversity and distribution of fragments input into the experiment. The barcoded MPRA and STARR-seq designs differ in whether DNA-seq and RNA-seq are carried out on barcodes associated with each query fragment (barcoded MPRAs) versus the query sequence itself (STARR-seq), but in both cases, the outcome variable of interest is the RNA-to-DNA ratio. This ratio captures a given fragment’s transcription rate, controlling for variation in query fragment abundance (Fig. 2). In barcoded MPRAs, each query fragment is associated with tens to hundreds of barcodes to ensure robustness and repeatability, while in STARR-seq several unique query fragments may overlap the same genomic location; as such, RNA-to-DNA ratios are often summarized for genomic windows a few hundred base pairs in size. In both categories of methods, the plasmid DNA library of interest is typically transfected into multiple pools of cells or tissues, such that DNA-seq and RNA-seq data are sourced from multiple technical replicates to assess reproducibility.

To identify regions of the genome with regulatory activity, researchers need to test whether a given fragment or genomic interval exhibits a significant excess of RNA relative to DNA. Several statistical methods have been proposed to accomplish this, for example, binomial tests [27] and differential peak calling approaches [57]. To relate a predictor variable of interest such as genotype, environment, or cell type to variation in RNA-to-DNA ratios, many researchers have relied on negative binomial [58] or linear model [59] pipelines originally developed for differential expression analyses. The best modeling approach will of course depend on the experimental details and the questions at hand, but several generalizable and flexible analysis pipelines are now available. For example, for barcoded MPRA experiments, mpralm [60] uses a linear modeling framework to test for differential activity, while MPRAanalyze [61] uses a graphical model to account for the uncertainty in both the DNA and RNA counts. For STARR-seq experiments, STARRPeaker [48] applies a negative binomial regression to identify regulatory elements.

Once the researcher has identified regions that show significant regulatory activity and/or differential regulatory activity as a function of some predictor of interest, several downstream analyses are possible for producing generalizable mechanistic insight. For example, significant regions can be tested for enrichment of transcription factor binding sites (e.g., using known vertebrate TF motifs [62]) or linked to nearby genes and tested for involvement in particular biological processes (e.g., using gene ontologies [63]). Significant regions can also be overlapped with other complementary taxa-specific datasets when available, for example summary statistics from GWAS, evolutionary or population genetic analyses, or other functional genomics assays.

## Current applications of MPRAs

Thus far, studies utilizing MPRAs have been largely focused on biomedical questions addressed in humans and model organisms. While a comprehensive review of all biomedical applications is beyond our scope (instead, see [53, 64–66]), we can briefly summarize this work as follows: MPRAs have been primarily used in biomedical research to tackle a long-standing question, what are the functional pathways linking non-coding regions to disease? MPRAs have shed light on this question by allowing researchers to (1) catalog enhancers, promoters [38, 67], and silencers [68] across a variety of disease-relevant human cell types [27, 69, 70] and cell states [41, 71–73] and (2) pinpoint causal alleles within broad disease-associated regions [36, 74]. Consequently, MPRAs have been extensively applied to help move beyond the vast GWAS catalogs generated in the past 15 years. For example, Choi and colleagues used a barcoded MPRA to characterize the effects of 832 variants in linkage disequilibrium with GWAS hits for melanoma. By pairing MPRA experiments with cis-eQTL mapping and colocalization analyses, the authors were able to identify 4 candidate variants that are likely causal to disease [75]. In another example, Inoue and collegues [76] used a lentiMPRA (Table 1) to characterize the dynamics of regulatory element activity across seven timepoints during early neural differentiation. This approach allowed the authors to identify temporally-dependent and independent TFs that regulate neuron development, and to reveal which elements are most active across time, including when cells occupy states of known importance for neurodegenerative disease. Through these studies and many other examples [64, 65, 77], MPRAs have proven their utility for uncovering the genetic and mechanistic basis of human disease.

A smaller but growing body of literature has applied MPRAs toward evolutionary questions. For example, MPRAs have been applied to study regulatory element evolution in primates [78] and *Drosophila* [28] by comparing the activity of homologous sequences across multiple species. These studies have identified individual regulatory sequences that have gained or lost activity across tens of millions of years of evolution, and have also pointed toward generalizable patterns that may characterize such changes. For example, Klein and colleagues recently linked CpG deamination to significant changes in regulatory element activity during primate evolution [78].

MPRAs have also been used to study the function of regions of putative significance to human evolution and human-specific traits. In one instance, Weiss and colleagues explored the effects of ~14k positions in the genome that diverged following the split between modern humans and archaic hominins (i.e., Neanderthals and Denisovans) [79]. By functionally assessing both the derived (modern human) and ancestral (archaic hominin) sequence for each region, they were able to show that 23% of regions that had *any* detectable regulatory activity also drove *differential* regulatory activity between modern humans and Neanderthals/Denisovans. These functionally differentiated sequences were enriched near genes involved in traits that also likely differed between modern and archaic humans, such as brain anatomy. Similarly, Uebbing and colleagues [80] as well as Whalen and colleagues [81] both assayed human accelerated regions in neural cell types. Whalen and colleagues coupled MPRA methods with human and chimp induced pluripotent stem cell (iPSC)-derived neural progenitors to compare human, chimpanzee, and intermediate/reconstructed ancestral sequences in equivalent cell types from both species. Using this comprehensive design, they showed that neuronal regulatory elements with consistent differences in human-chimp activity are almost completely dependent on *cis*-regulatory sequence, with little evidence for interaction with the *trans*-acting cellular environment. Finally, MPRAs have been used to understand the functional consequences of archaic admixture. Jagoda and colleagues [82] as well as Findley and colleagues [83] quantified the regulatory activity of variants introgressed from Neanderthals into the modern human gene pool. Both studies found that the *in vitro* activity of many of these variants was suggestive of causal effects on gene regulation. The authors therefore hypothesized that Neanderthal-introgressed variants contribute to phenotypic variation today through altered transcriptional regulation. It will be exciting to see if additional follow-up work (e.g., in *in vivo* models) confirms these results.

## Expanding MPRA usage in evolutionary biology

**Fig. 3:**
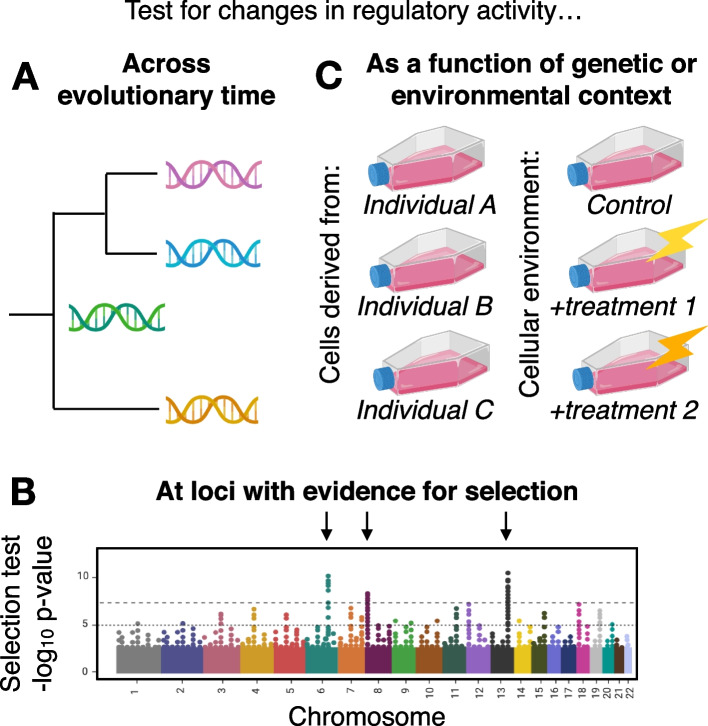
Study designs for evolutionary questions. **A** MPRAs can be used to test for changes in regulatory activity across evolutionary time, by assaying orthologous sequences across a phylogeny (pink, blue, and yellow tip lineages) and/or using ancestral sequence reconstruction to assay sequences from extinct taxa (green lineage). **B** MPRAs can be used for fine mapping of functional alleles identified through sequence-based scans for positive selection. **C** MPRAs could be used to understand how genetic interactions, namely epistasis and genotype-by-environment interactions, impact regulatory variation. This could be accomplished by assaying a genetically variable MPRA library across *trans* cellular backgrounds that are either genetically or environmentally diverse

The examples above highlight the power of MPRAs for improving our understanding of the evolution of phenotypic diversity. While such work so far has been limited to humans and select other taxa, it is highly feasible to apply these approaches to a broader range of species. By applying MPRAs to diverse study designs and organisms, including non-model organisms, many outstanding evolutionary questions could be answered. For instance, in combination with ancestral sequence reconstruction approaches, MPRAs make it possible to test regulatory elements for changes in activity across evolutionary time. In other words, it is possible to assay sequences from both extant and extinct taxa, and thus to explore the evolution of gene regulation in general, as well as specific regulatory element-controlled organismal traits (Fig. 3A). The strength of this particular approach is unavoidably reliant on the quality and number of existing genome assemblies and is thus not well suited to sparsely sampled phylogenies (we also note there are some caveats in reconstructing ancestral states [84], especially of sequences under selection [85]). However, as the breadth and depth of sequenced genomes increases—for example, through large-scale initiatives such as the Vertebrate Genomes Project, Earth Biogenome Project, and DNA Zoo [86, 87]—this approach will become more generalizable.

Another possibility is to use MPRAs for fine mapping of functional alleles identified through sequence-based scans for positive selection (Fig. 3B), analogous to their use to fine-map eQTLs [36] or GWAS hits [74, 75]. This could be accomplished by independently testing all SNPs in high linkage disequilibrium within an outlier region or score peak, or, potentially, by tiling across longer elements in small steps to identify functional modules such as key TF binding sites. A key component of such a design would be a set of control, neutrally evolving regions included in the input library for comparison and for developing background expectations. Together, this sort of approach would be extremely useful for addressing a long-standing challenge in evolutionary and population genomics: linking sequence-based measures of adaptation to molecular function and mechanism.

MPRAs could also be applied to understand how genetic interactions (i.e., epistasis and genotype-by-environment interactions) impact phenotypic variation. Notably, genetic interactions have long been thought to be important for complex trait evolution, yet they are notoriously difficult to study because traditional approaches require very large sample sizes to reach statistical robustness [88, 89]. MPRAs can be used to make progress in this area. For example, one could assemble a library that includes multiple genotypic versions of a given set of regulatory elements, and then systematically test it (1) within a cell line exposed to different environmental perturbations, (2) within cell lines representing different tissues, or (3) within cell lines derived from the same tissue but from individuals of different genetic backgrounds or species (Fig. 3C). Doing so would generate quantitative estimates of how varying contexts interact with genetic variation to impact regulatory element activity with unprecedented flexibility and resolution.

Importantly, some groundwork has already been laid for these types of study designs. In their study of human-specific variants, for example, Weiss and colleagues tested three different cell types—pluripotent stem cells, osteoblasts, and neural progenitors—and found that most variants were only differentially active between modern and archaic hominins in one of the three cell types [79]. In another example, van Arensbergen and colleagues generated genome-wide MPRA libraries for four individuals included in the 1000 Genomes Project: one person each of Punjab, Japanese, Puerto Rican, and Mende ancestry [90]. Across all four individuals, they found ~30k SNPs that significantly altered regulatory activity in K562 cells (a leukemia cell line), HepG2 cells (a hepatocarcinoma cell line), or both cell types. Together, these studies provide preliminary evidence for genotype-by-environment effects (in the form of genotype-by-cell type effects), at least in humans. We see great potential for expanding this type of work to other species and other types of genetic interactions.

The above examples highlight how MPRAs can be used to catalog the impact of both extinct and extant variation within a population or species at scale. In parallel, deep mutational scans have recently moved beyond a focus on known genetic variation to cataloging the effects of all possible mutations within a genomic feature. Taking advantage of error-prone PCR, Kircher and colleagues tested 99.9% of all possible SNPs across 20 different disease-associated regulatory elements to identify those most likely to contribute to their pathogenicity [38]. They found that sequence-based scores of phenotypic impact were generally poor predictors of regulatory activity, pointing to the necessity of functional assays for understanding the consequences of disease-associated variants. To our knowledge, these sorts of approaches have not been applied at a comparable scale to loci of evolutionary interest, although nothing inherently precludes doing so. Such approaches would be extremely useful for understanding the genotype-phenotype relationship and the landscape of putatively adaptive mutations.

## Challenges and recommendations for expanded usage

There are several reasons why MPRA usage has been largely restricted to humans and model organisms thus far. First, we believe there is limited awareness of MPRAs in ecology and evolutionary biology communities, which was a main motivator for writing this review. Second, MPRAs are complex assays and require access to specialized equipment, and more generally, access to specialized know-how to design, carry out, and analyze. However, most of the equipment (e.g., biosafety cabinets, incubators, electroporators) is common in molecular- or genetics-focused departments and likely already exists at most institutions. Further, several detailed MPRA protocols and analysis pipelines [37, 48, 60, 61] are now publicly available [37, 40, 91] (Additional file 1: Table S1), making it increasingly feasible for researchers from diverse disciplines to apply these assays. Finally, in addition to specialized equipment and know-how to carry out MPRA experiments, these approaches also require (1) a high-quality genome sequence and/or large amounts of genetic material, depending on the study design; (2) a relevant primary cell pool or immortalized cell line for transfection; and (3) a working knowledge of potential interpretative challenges. These constraints have likely hindered the widespread adoption of MPRAs; below, we discuss how they can be overcome.

The barcoded MPRA design typically relies on large-scale oligosynthesis of known genomic sequences, and thus a reference genome is required. Reference genomes are increasingly available for most study organisms, as well as increasingly feasible to generate *de novo* [92, 93]. Alternatively, a subset of the genome could be sequenced at a much lower cost using methods like RAD-seq [94, 95], as well as methods that specifically target gene regulatory elements (e.g., ChIP-seq [96, 97] or ATAC-seq [44]), which can then be used to refine the list of testable sequences. A more general challenge for barcoded MPRAs is that oligosynthesis is limited in both capacity and sequence length: commercial providers rarely synthesize fragments longer than 300 bp. This means that most barcoded MPRAs test short sequences, or require sliding window designs to examine larger ones, introducing additional complexity during analysis. While 300 bp is enough to capture, for example, specific TF binding sites and local interactions, many complete regulatory elements are larger than 300 bp. Indeed, studies thus far demonstrate increased power to detect regulatory activity when query fragments are larger, as well as a general impact of fragment length on downstream assay output [46, 53].

An alternative approach is to use STARR-seq family methods (Table 1) to support testing of larger fragments. Such approaches can leverage sequence capture or other methods to target DNA fragments of interest, as well as random shearing to cover an entire genome. Either design requires access to large amounts of starting genetic material (e.g., a few [27] to hundreds [71] of micrograms of DNA, or potentially reliance on whole genome amplifications [98]); this input requirement may pose challenges when working with rare samples or endangered species. However, once a plasmid library is generated, it can be easily renewed via bacterial transformation with minimal loss of diversity [46]. Therefore, while it may be challenging to collect micrograms of DNA for some species, for many study designs, this obstacle only needs to be overcome once; the resulting plasmid library can then support multiple experiments and even be shared across the scientific community. Depending on the questions, it may also be worthwhile to pool smaller amounts of material from many individuals to create a single library of genetically diverse regulatory elements [42].

Once a plasmid library is assembled, an unavoidable challenge for many studies will be the need for a cell line that can be grown at scale, efficiently transfected (or infected), and is representative of the species and tissue of interest. The first two requirements are intimately linked to the number of sequences that can be tested in a given assay. This is because each sequence of interest must be assayed independently multiple times to achieve robust statistical power. Recent recommendations in the field for barcoded MPRA designs are to ensure that every sequence is represented by 50–100 independent barcodes, with multiple observations of each barcode [36]. With these numbers, testing just 20,000 sequences with standard designs may require transfection of 10–20 million cells, with larger starting cell amounts needed since transfection efficiency is never 100%. For STARR-seq designs, recommendations are to successfully transfect ~60 or ~300 million cells for focused versus genome-wide screens, respectively [40].

These cell numbers can be prohibitive in the case of hard-to-transfect, terminally differentiated, or non-proliferative cell types, or when working with rare samples or non-model species. Indeed, commercially available cell lines with pre-optimized growth and transfection protocols are for the most part limited to humans and model organisms, though a growing number of commercially available products are available for other species (see Additional file 1: Fig S2 for a complete list [99]). In some cases, it may be feasible to use modified MPRA protocols appropriate for hard-to-transfect cell types and/or limited cell quantities [81, 100–102], or to derive new cell lines for non-model species [103]. In other cases, a better solution may be to use a cell line from a closely related species as a proxy (e.g. [28, 78],). This design assumes a conserved *trans* environment since the split of the focal and cell line species, but there is strong evidence that TF expression, structure, and specificity to binding motifs are well-conserved across long evolutionary time scales [104–106]. For instance, we reanalyzed gene expression data from human, gorilla, chimpanzee, orangutan, and macaque lymphoblastoid cell lines [107–109] (LCLs) and compared TF expression levels between humans and each of the other species. We found that TF expression levels in LCLs are highly conserved across species pairs spanning ~6 to ~26 million years of evolutionary divergence (R^2^ for pairwise comparisons=0.66-0.76; Additional file 1: Fig S3). It is also worth highlighting that one MPRA study so far, in humans and chimpanzees, has already shown that the overwhelming majority of human-chimpanzee species differences in regulatory element activity arise from the query fragment sequence itself rather than the species-specific cellular environment; in this study, *trans* effects generated differences in activity for <1% of regulatory elements [110]. Thus, several lines of evidence suggest that the easiest solution for non-model organism researchers is to use an existing cell line from a closely related species and that this choice will have minimal effects on evolutionary inferences.

Finally, we caution that there are still interpretive challenges with MPRA data, as there are with any functional assay, and evolutionary researchers must be aware of these caveats. First, a fragment’s regulatory activity will always be specific to the cell type it was assayed in, and in some cases, a lack of regulatory activity may simply indicate that the relevant cell type was not used, rather than that the fragment is not important. Extreme cell type specificity is likely to be the exception rather than the rule, but this is still a key consideration especially if the relevant cell type is not known *a priori*. Second, recent studies have found that the type of promoter included in the MPRA plasmid can affect a fragment’s regulatory activity. For example, thousands of regions in the *Drosophila melanogaster* genome exhibited differential regulatory activity when the STARR-seq vector was redesigned to include a developmental versus a housekeeping promoter [111]. The standard versions of both the STARR-seq and barcoded MPRA vectors include a super core promoter that is designed to be generally active and to interact with a broad range of elements. Unless researchers are interested in promoter-enhancer specificity, we recommend sticking with the standard versions (plasmids #99296 and #71499 in Addgene). Third, there is currently no consensus on what counts as a biologically meaningful effect size in MPRA studies. This interpretive challenge is of course not unique to MPRA studies, but given that many fragments are typically assayed in a small number of replicates followed by multiple hypothesis testing correction, we actually suspect that the MPRA literature includes more false negatives than false positives. Another approach, popular with barcoded MPRAs, is to include control sequences (i.e., known regulatory elements) to which query fragments can be compared. As MPRA technologies are more broadly applied, mindful interpretation of the data will continue to be a key discussion for the field.

## Potential future directions and conclusions

Moving forward, we speculate that there are two areas where emerging research from non-evolutionary fields will hopefully soon benefit evolutionary biologists, and in turn catalyze research in this area. First, there is a growing awareness of the potential MPRAs hold, and a growing community drive to develop standards to facilitate community adoption and data reuse. For instance, MaveDB provides a resource for deposition of results from MPRAs (and other types of MAVEs) under a standardized format [112]. Similarly, the nascent Alliance of Variant Effects (AVE) seeks to build an atlas of all possible variants in disease-related functional elements in the human genome [113]. These existing data collections could be mined for inferences about human evolution, but more broadly, these efforts signal that public, standardized databases will be the norm going forward, and will surely benefit the evolutionary community as they are expanded to a wider range of species. Second, MPRAs have recently motivated new bioinformatic and statistical tool development [48, 60, 61, 114, 115] , which could aid non-model organism researchers as more MPRA data are generated for these species. For example, MPRA data can be coupled with machine learning approaches [81, 116–125] to predict gene expression and regulatory structure from genomic sequence alone. MPRA-DragoNN [116] and DeepSTARR [117] both use convolutional neural networks to learn sequence features associated with regulatory element activity. These tools could allow non-model organism researchers to bioinformatically generate genome-wide regulatory maps from a focused MPRA training dataset, or potentially, from one generated for a closely related species.

Like most other genomic technologies, MPRAs were first optimized in systems with extensive genomic resources (i.e., humans and model organisms). However, for evolutionary biologists, these approaches often become most exciting once they are expanded to a more diverse set of species and contexts—even if these extensions come with caveats and challenges. We believe that the biological insights to be gained from applying MPRAs to diverse organisms, environments, and study designs have substantial potential for addressing evolutionary questions. In particular, we believe MPRAs will soon expand our ability to interpret and annotate the genomes of non-model organisms, as well as our understanding of how gene regulation contributes to adaptive evolution and phenotypic diversity. The already demonstrated significance of MPRAs in the biomedical sciences suggests that, in the coming years, we can expect an equivalent wealth of insights drawn across a broad range of taxa and evolutionary questions.

## Supplementary Information

**Additional file 1.** Supplementary Methods, Figs S1-S3, Table S1 and Supplementary References.

**Additional file 2.** Review history.
