# Supplementary material for: Leveraging massively parallel reporter assays for evolutionary questions
Source: Genome Biol. 2023 Feb 14;24:26. doi: 10.1186/s13059-023-02856-6 (PMC9926830; doi:10.1186/s13059-023-02856-6)
Supplement: Supplementary file 1 — Additional file 1. Supplementary Methods, Figs S1-S3, Table S1 and Supplementary References. [file 13059_2023_2856_MOESM1_ESM.docx]

Supplementary Materials for Gallego Romero and Lea, “**Leveraging massively parallel reporter assays for evolutionary questions**”

This PDF contains:

Supplementary Methods

Figure S1

Figure S2

Figure S3

Table S1

Supplementary References

**Supplementary Methods**

To understand the existing cellular resources for non-human species, we downloaded the ATCC catalog for all animal species excluding humans. We then used the R package taxize^1^ to download taxonomic hierarchical information for each species using the option for querying the NCBI database. In a few cases, we corrected spelling errors or out of date genus names to recover taxonomic information.

To understand how conserved TF expression is through evolutionary time, we reanalyzed gene expression data from human, gorilla, chimpanzee, orangutan, and macaque lymphoblastoid cell lines^2^ and compared TF expression levels between humans and each of the other species. To do so, we used the R package biomaRt^3^ to identify orthologous protein coding genes for each human and non-human primate species pair. We calculated the species-specific mean log_2_ TPM value and removed genes that were not expressed in either species. Finally, we compared gene expression levels across all protein coding genes as well as for TF genes alone using Pearson’s R^2^. Results are plotted in Figure S1.

All analyses were conducted in R version 4.1.2. Select figures use cartoon images from BioRender.com.

**Supplementary Figures**

**Fig S1. Hierarchy of terminology used in this review**. Terms in blue are the focus of this review.

**Fig S2. Animal species currently represented in the American Type Culture Collection (ATCC) catalog.** Phylogenetic tree of all species with entries in the ATCC catalog, color coded by the number of entries. Select species are represented with cartoons to orient the reader to where broad taxonomic groups fall on the phylogeny.

**Fig S3. Conservation of transcription factor (TF) expression across primate species**. x and y-axes represent the mean log_2_ gene expression values for a given species (in terms of transcripts per million, TPM). Only orthologous genes for a given species pair are included, and each dot represents a gene (colored according to whether it is or is not a TF in the TRRUST database of mammalian transcription factors^4^). Dotted lines represent x=y.

**Table S1. Available step by step protocols.**

| **Assay** | **Protocol link** |
| --- | --- |
| ATAC-STARR-seq | https://www.protocols.io/view/atac-starr-seq-5jyl89rorv2w/v1 |
| STARR-seq and UMI-STARR-seq | https://currentprotocols.onlinelibrary.wiley.com/doi/full/10.1002/cpmb.105 |
| mSTARR-seq | http://www.tung-lab.org/protocols-and-software.html |
| CapSTARR-seq | https://protocolexchange.researchsquare.com/article/nprot-4333/v1 |
| Barcoded MPRA | https://www.protocols.io/view/massive-parallel-reporter-assay-mpra-kxygxpmkwl8j/v1 |
| Barcoded MPRA | http://noonan.ycga.yale.edu/noonan_public/Uebbing_Gockley_MPRA/Extended_Methods.pdf |
| lentiMPRA | https://www.nature.com/articles/s41596-020-0333-5 |

**Supplementary References**

1. Chamberlain, S. A. & Szöcs, E. taxize: taxonomic search and retrieval in R. F1000Res. 2, 191 (2013).
2. García-Pérez, R. et al. Epigenomic profiling of primate lymphoblastoid cell lines reveals the evolutionary patterns of epigenetic activities in gene regulatory architectures. Nat. Commun. 12, 3116 (2021).
3. Durinck, S., Spellman, P. T., Birney, E. & Huber, W. Mapping identifiers for the integration of genomic datasets with the R/Bioconductor package biomaRt. Nat. Protoc. 4, 1184–1191 (2009).
4. Han et al. TRRUST v2: an expanded reference database of human and mouse transcriptional regulatory interactions. Nucleic Acids Res. 46, D380–D386 (2018).
