# Supplementary material for: Leveraging massively parallel reporter assays for evolutionary questions
Source: Genome Biol. 2023 Feb 14;24:26. doi: 10.1186/s13059-023-02856-6 (PMC9926830; doi:10.1186/s13059-023-02856-6)
Supplement: Supplementary file 2 — Additional file 2. Review history. [file 13059_2023_2856_MOESM2_ESM.docx]

Review History

**First round of review**

**Reviewer 1**

**Comments to author:**

The review by Romero and Lea provides a concise but comprehensive overview of high-throughput reporter assays aimed at researchers who work with non-model organisms and/or questions related to cross-species effects of cis-regulatory elements. The authors do a wonderful job describing reporter assays. This is not an easy task given the field is littered with acronyms and one-off assays. Furthermore, they provide an excellent overview of how the assays have been applied previously. I am an avid reader of all papers that involve these assays and the authors even managed to identify several papers that were new to me that are now on my reading list!

Overall the review is very well written and the structure of the review is logical. I have several comments below. It is important to note, most of the items below have some subjective component to them. That is, they reflect a personal opinion or viewpoint of mine that I provide as a suggestion to the authors towards improving the review.

1. The term "enhancer" is used on pg3:line20 and elsewhere in the review. I would consider using terminology that is more inclusive to the range of functions regulatory elements can have. Cis-regulatory element is one suggestion but there are other options the authors could consider.

2. I would include Melnikov et al 2012 (PMID: 22371084) as a citation when describing the first MPRAs reported in the literature on pg4:line50.

3. The authors use MPRA to describe all high-throughput reporter assays (e.g. STARR, pGL4 vectors etc) and as a term to specify assays where only the barcode is captured in the 5'/3' UTR (usually with a pGL4 backbone). This latter definition is the one I typically use; however I recognize that some groups include STARR as a subassay of MPRA. Regardless, I did find the switching between usage in the review confusing at times and would suggest a different term to differentiate all high-throughput reporter assays and assays where only a barcode is captured in the UTR.

4. Pg5:line37 Traditional MPRAs (pGL4 vectors) technically can also use selected fragments. I would suggest changing "Additionally" to "As a result, STARR-seq is more economical for testing large libraries such as captured, immunoprecipitated….."

5. Figure 1A, I would add a line from the plasmid in step 4 to steps 7-8. The final plasmid library is the plasmid used for quantification. The plasmid in step 3 is used only for barcode assignments when barcodes are added post synthesis.

6. Figure 1B, I would add a line from step 2 to the quantification step since a critical step in the quantification process is normalization of the signal tracks based on the input library.

7. Pg8:line15. I would temper this sentence and describe these variants as being variants that show promising characteristics for functional follow up. Personally, I view MPRA as an excellent screening tool that provides a compelling piece of evidence that a variant may have a functional effect in the genome. The false positives and negatives are real issues with MPRA that researchers should be aware of when undertaking a study using the assay.

8. Pg9:line34. I would remove the discussion about the van Arensbergen study. The actual number of variants the authors were able to confidently make allele specific measurements on is between 300K-400K (the union is not clear in the manuscript but the number for each cell type is provided in the methods). Furthermore, their conclusion regarding cell-type specificity is likely driven by technical issues where you have a winner's curse effect in the discovery cell type and are then underpowered when looking at replication in a different cell type. I am not aware of a study where a proper comparison between cell types has been performed (e.g. directly testing if cell type A is different relative to cell type B instead of simply asking if cell type A or B is different than zero). Weiss and colleagues likely ran into similar technical issues but refrained from directly exploring cell type specificity probably for this exact reason.

9. Table 1: Newer versions of STARR-seq use the ORI as the promoter, not a minimal promoter.

**Reviewer 2**

**Comments to author:**

This review covers current efforts and challenges in using Massively Parallel Reporter Assays (MPRAs) and related protocols in addressing questions of evolutionary significance. The authors first note that MPRA has been, to-date, deployed mostly for biomedical research questions. However, given the importance of gene-regulation across a number of evolutionary processes, the authors note the potential of MPRA in evolutionary studies. The authors continue with an outline of current MPRA and MPRA-related protocols, including "classic" MPRA, STARR-seq, lentiMPRA, and saturation-mutagenesis MPRA. They progress to describe the potential scope of MPRA in evolutionary studies, including assaying genetic sequences from different timepoints in the evolutionary timescale, testing variants associated with positive selection and selective sweeps, and testing the effect of genetic interactions in complex trait evolution. Challenges in using MPRA for evolutionary studies are then described, such as oligosynthesis barriers limiting the size of assayable DNA regions, the still incomplete encyclopedia of genomes across the tree of life, and technical challenges with specific cell types/species. The authors conclude with a perspective in how MPRA can lead to technical innovations/standardization in bioinformatics/statistics and how this assay can shed light on the evolutionary dynamics of non-model organisms.

Major

-'DNA sequences of interest are synthesized, each in conjunction with a unique barcode' many current protocol insert the barcodes when PCRing the oligos, so would revise this to be more general to 'DNA sequences of interest are synthesized, cloned in conjunction with a unique barcode into a specially engineered plasmid'. Also, 'Second, a basal promoter and a reporter gene are inserted between the sequence of interest and the barcode, such that the barcodes reside in the 3'UTR of the reporter gene' there are many protocols that after barcodes are inserted after oligo PCR the sequences are then just cloned into the MPRA vector. In addition, barcodes are also placed in the 5'UTR, in particular for lentivirus-based MPRA to reduce issues with lentivirus recombination (lentivirus packages 2 RNA strands that love to recombine). Finally, another key step is to associate the barcodes to sequences if done via PCR. Also, there are many versions that use AAV or lentivirus to do MPRA, so would not just have 'transfected'. So in sum, to be more general here, would revise this whole section to something like:

'DNA sequences of interest are synthesized, cloned in conjunction with a unique barcode into a specially engineered plasmid that contains a basal promoter and a reporter gene. If barcodes are added during the cloning step, the library is sequenced to associated barcodes to the assayed sequence. Second, the reporter library is transfected or infected, if virus is used for its delivery, into a cell type…'

Would revise figure accordingly also for all of these including delivery methods.

-Figure 1 is hard to follow and needs a lot of work to simplify it and make it easier for a reader to get. In addition to comments above, would remove things that are not needed to simplify. For example, would take out polyA signal. Would change 'Promoter' to 'basal promoter' or 'minimal promoter', would make ORF (GFP) green color. Would make barcodes have some vertical lines in them to look like barcodes. Would take out plasmid from the circle that is a cell and just draw a cell without these. It is quite confusing as is. Would write RNA/DNA in y-axis for MPRA. Would run this figure through people who do not know MRPA/STARR-seq and see if they got it and get input from other published figures also.

-'Additionally, STARR-seq allows the researcher to use captured, immunoprecipitated, or otherwise selected genomic DNA fragments, as well as randomly fragmented DNA' This is incorrect, as MPRA also allows that. Would instead mention that for both sequences can be either oligosynthesized, PCRed, captured or immunoprecipitated. This also needs to be corrected later when the authors mention 'An alternative approach is to use STARR-seq family methods' as again MPRA can and has used these cloning approaches also. Also, while he advantages of STARR-seq relative to classic MPRA are mentioned, the converse is not. For example, the multiplicity of barcodes associated with each candidate enhancer in MPRA design shields it from the sequence-specific mRNA stability defects present in STARR-seq. Both techniques have limitations and advantages and this should be accurately represented. As mentioned also below in minor comments, would be good to reference the reader to PMID 33046894 (reference 84) which compared 9 different MPRA and possibly discuss this a bit with the limitations of each method and how comparable they were from this article (standard transfection, ORI STARR-seq and lentiMPRA all correlated well to one another and each had various limitations) sending the reader to this article for more detail.

-Line 57, page 4: DNA-seq is also a readout of MPRA, which is necessary for normalizing the RNA-seq output by the transfection efficiency of each tested candidate enhancer. Similarly, in Figure 1B, DNA-seq should be included in the STARR-seq workflow. Would also provide more general text on how MPRA or STARR-seq measurements are done. What is used to determine if a sequence is active. Can also reference tools to do this, such as MPRAflow, MPRAanalyze, STARRPeaker and others. This will be helpful for readers who might be fearful of this technology that the authors are trying to entice, that also the computational tools are available for it.

-In the 'These cell numbers can be prohibitive in the case of hard-to-transfect' paragraph, another option could also be carrying out MPRA in the organism of interest. MPRA was done in mice, via hydrodynamic tail vein injections or AAV, flies, ciona and could potentially be developed in other organisms. Would add text to mention this as another approach.

-There are several mentions of using MPRA to study phenotypic evolution and divergence. While MPRA is described succinctly, a description of how data from an MPRA-style experiment can then be linked to target genes, traits and phenotypes is lacking.

-Figure 3 feels more like a supplemental figure. Similar to Figure 2, would maybe have instead for Figure 3 in this section the various recommendations for expanded usage.

Minor

-Would remove 'in vitro' in abstract from 'single in vitro experiment' as MPRAs have also been carried out in vivo.

-Would remove 'explicitly' in 'to address explicitly evolutionary'

-'although often within 1 megabase7,10'as some genomes discussed here will be much smaller, to not confuse readers, would mention for what genomes this work determined with 1 megabase.

-Line 25 on page 3: the author mentioned that enhancer regulates transcription by recruiting RNA polymerase II (Pol II). Though Pol II can be recruited by some enhancers, it's mostly recruited to promoters near transcription start sites.

-Line 28 on page 3: change 'Enhancers' to 'Candidate enhancers', as the number of functionally validated enhancers is far less than the number of protein-coding genes.

-Line 55 on page 4: change 'to a unique barcode' to 'unique barcodes'. In most cases, a sequence tested in MPRA is linked to multiple barcodes.

-'This is largely because they are difficult to identify from genomic or epigenomic datasets' I would also add another important hurdle here that enhancers are tissue/cell type specific and also context or temporal specific, i.e. can only be on if due to some perturbation or at a certain developmental time point for example. Due to these they are also hard to identify.

-'lead to a reduction' change to 'leads to a reduction'.

-' these changes to the design impact the types of information that can be gained from a given assay (Table 1).' Might want to finish this sentence with some words referencing PMID 33046894 which compared 9 different MPRA techniques to send the reader to read more and understand what method might be good for them.

-'Further, several detailed MPRA protocols are now publicly available75,76' Would also add PMID 32641802 which provides a detailed protocol (reference 38).

-In the section named 'CURRENT APPLICATIONS OF MPRAS', most discussions were focused on MPRA studies in the evolutionary field. Thus, it is more appropriate to name the section 'CURRENT APPLICATIONS OF MPRAS IN EVOLUTION'. Also, since the current applications of MPRAs were just briefly touched up here, it is necessary to mention that this is beyond the scope of this article and refer readers to other more comprehensive reviews of MPRA.

-Line 47 on page 7: the 14k SNPs are found in modern humans but not in archaic hominins and great apes. The author didn't mention great apes.

-Since section 'EXPANDING MPRA USAGE IN ECOLOGY AND EVOLUTION' mostly talked about things that could be done in the future. It would be better to combine this section with 'FUTURE DIRECTIONS AND CONCLUSIONS.

-In Figure 2 weird to have B below and C next to A. Would move things around.

**Reviewer 3**

**Comments to author:**

This is a timely and useful review of MPRA technology and its potential use to address evolutionary questions. The authors nicely summarize recent developments in this field, and correctly point out that advances in biomedical applications of MPRAs can just as easily be transferred to evolutionary questions. The review clearly describes MPRA technology, cites major references (the field is much too large to cite comprehensively in a short review), and provides helpful tables to guide readers unfamiliar with the technology.

While the authors discuss some of the technical challenges of implementing MPRAs, this review would benefit from a short discussion of the limitations and interpretive challenges of MPRA data. The authors note the importance of cell type, which is a critical limitation when MPRAs are used to test SNPs function. Often the correct cell type is not only unavailable, but also unknown when candidate SNPs and cis-regulatory elements are selected from sequence alone, as in GWAS, cross-species comparisons, human accelerated regions, etc. The choice of basal promoter can determine which elements are active in the assay. If a cis-regulatory element with a potentially important SNP is inactive in the assay, the effect of the SNP cannot be assessed. There is also a question of what counts as a meaningful effect size for a variant in an MPRA. In some cases MPRAs are able to detect small effect sizes, but it is not clear whether those are phenotypically meaningful. On the other hand, given that cellular context is important, it is not clear that SNPs with the largest effects in MPRAs are the most functionally relevant within their genomic and cellular context.

Related to these issues are questions of what controls to include in MPRAs. For example, when using MPRAs to fine map alleles from scans for positive selection, should the MPRA library include a control set of alleles from neutrally evolving loci in order to estimate the assay's false positive rate?

MPRAs are a powerful technology, but interpretation of the results is an important issue that should receive more emphasis.

**Authors Response**

**Point-by-point responses to the reviewers’ comments:**

**Comments from reviewer 1:**

The review by Romero and Lea provides a concise but comprehensive overview of high-throughput reporter assays aimed at researchers who work with non-model organisms and/or questions related to cross-species effects of cis-regulatory elements. The authors do a wonderful job describing reporter assays. This is not an easy task given the field is littered with acronyms and one-off assays. Furthermore, they provide an excellent overview of how the assays have been applied previously. I am an avid reader of all papers that involve these assays and the authors even managed to identify several papers that were new to me that are now on my reading list! Overall the review is very well written and the structure of the review is logical. I have several comments below. It is important to note, most of the items below have some subjective component to them. That is, they reflect a personal opinion or viewpoint of mine that I provide as a suggestion to the authors towards improving the review.

Thank you for your kind words and support of our work!

1. The term "enhancer" is used on pg3:line20 and elsewhere in the review. I would consider using terminology that is more inclusive to the range of functions regulatory elements can have. Cis-regulatory element is one suggestion but there are other options the authors could consider.

We agree. We’ve edited to use the more general term of “regulatory element” where appropriate. In some cases, we are defining particular properties of enhancers and therefore still use this term as needed.

2. I would include Melnikov et al 2012 (PMID: 22371084) as a citation when describing the first MPRAs reported in the literature on pg4:line50.

Citation added.

3. The authors use MPRA to describe all high-throughput reporter assays (e.g. STARR, pGL4 vectors etc) and as a term to specify assays where only the barcode is captured in the 5'/3' UTR (usually with a pGL4 backbone). This latter definition is the one I typically use; however I recognize that some groups include STARR as a subassay of MPRA. Regardless, I did find the switching between usage in the review confusing at times and would suggest a different term to differentiate all high-throughput reporter assays and assays where only a barcode is captured in the UTR.

To avoid confusion, we’ve changed the terminology to use “MPRA” for all high-throughput reporter assays and “barcoded MPRA” to specify assays where only the barcode is captured in the 5'/3' UTR.

4. Pg5:line37 Traditional MPRAs (pGL4 vectors) technically can also use selected fragments. I would suggest changing "Additionally" to "As a result, STARR-seq is more economical for testing large libraries such as captured, immunoprecipitated….."

Thanks, we’ve made this edit.

5. Figure 1A, I would add a line from the plasmid in step 4 to steps 7-8. The final plasmid library is the plasmid used for quantification. The plasmid in step 3 is used only for barcode assignments when barcodes are added post synthesis.

Added.

6. Figure 1B, I would add a line from step 2 to the quantification step since a critical step in the quantification process is normalization of the signal tracks based on the input library.

Added.

7. Pg8:line15. I would temper this sentence and describe these variants as being variants that show promising characteristics for functional follow up. Personally, I view MPRA as an excellent screening tool that provides a compelling piece of evidence that a variant may have a functional effect in the genome. The false positives and negatives are real issues with MPRA that researchers should be aware of when undertaking a study using the assay.

This is a very fair point, and we’re sensitive to the fact that MPRA analysis pipelines are not straightforward and can result in false positive and false negative issues. We’ve tempered the sentences as suggested.

8. Pg9:line34. I would remove the discussion about the van Arensbergen study. The actual number of variants the authors were able to confidently make allele specific measurements on is between 300K-400K (the union is not clear in the manuscript but the number for each cell type is provided in the methods). Furthermore, their conclusion regarding cell-type specificity is likely driven by technical issues where you have a winner's curse effect in the discovery cell type and are then underpowered when looking at replication in a different cell type. I am not aware of a study where a proper comparison between cell types has been performed (e.g. directly testing if cell type A is different relative to cell type B instead of simply asking if cell type A or B is different than zero). Weiss and colleagues likely ran into similar technical issues but refrained from directly exploring cell type specificity probably for this exact reason.

Thanks for pointing this out, we’ve removed the discussion about cell-type specificity.

9. Table 1: Newer versions of STARR-seq use the ORI as the promoter, not a minimal promoter.

We’ve edited to include a sentence about the new version of the STARR-seq vector.

**Comments from reviewer 2:**

This review covers current efforts and challenges in using Massively Parallel Reporter Assays (MPRAs) and related protocols in addressing questions of evolutionary significance. The authors first note that MPRA has been, to-date, deployed mostly for biomedical research questions. However, given the importance of gene-regulation across a number of evolutionary processes, the authors note the potential of MPRA in evolutionary studies. The authors continue with an outline of current MPRA and MPRA-related protocols, including "classic" MPRA, STARR-seq, lentiMPRA, and saturation-mutagenesis MPRA. They progress to describe the potential scope of MPRA in evolutionary studies, including assaying genetic sequences from different timepoints in the evolutionary timescale, testing variants associated with positive selection and selective sweeps, and testing the effect of genetic interactions in complex trait evolution. Challenges in using MPRA for evolutionary studies are then described, such as oligosynthesis barriers limiting the size of assayable DNA regions, the still incomplete encyclopedia of genomes across the tree of life, and technical challenges with specific cell types/species. The authors conclude with a perspective in how MPRA can lead to technical innovations/standardization in bioinformatics/statistics and how this assay can shed light on the evolutionary dynamics of non-model organisms.

This was an excellent summary.

'DNA sequences of interest are synthesized, each in conjunction with a unique barcode' many current protocol insert the barcodes when PCRing the oligos, so would revise this to be more general to 'DNA sequences of interest are synthesized, cloned in conjunction with a unique barcode into a specially engineered plasmid'. Also, 'Second, a basal promoter and a reporter gene are inserted between the sequence of interest and the barcode, such that the barcodes reside in the 3'UTR of the reporter gene' there are many protocols that after barcodes are inserted after oligo PCR the sequences are then just cloned into the MPRA vector. In addition, barcodes are also placed in the 5'UTR, in particular for lentivirus-based MPRA to reduce issues with lentivirus recombination (lentivirus packages 2 RNA strands that love to recombine). Finally, another key step is to associate the barcodes to sequences if done via PCR. Also, there are many versions that use AAV or lentivirus to do MPRA, so would not just have 'transfected'. So in sum, to be more general here, would revise this whole section to something like: 'DNA sequences of interest are synthesized, cloned in conjunction with a unique barcode into a specially engineered plasmid that contains a basal promoter and a reporter gene. If barcodes are added during the cloning step, the library is sequenced to associated barcodes to the assayed sequence. Second, the reporter library is transfected or infected, if virus is used for its delivery, into a cell type…'

Thanks for mentioning this, we’ve edited this text as suggested.

Would revise figures accordingly also for all of these including delivery methods.

Good point. We’ve added text to the legend of Figure 1 to make it clear there are multiple delivery methods.

Figure 1 is hard to follow and needs a lot of work to simplify it and make it easier for a reader to get. In addition to comments above, would remove things that are not needed to simplify. For example, would take out polyA signal. Would change 'Promoter' to 'basal promoter' or 'minimal promoter', would make ORF (GFP) green color. Would make barcodes have some vertical lines in them to look like barcodes. Would take out plasmid from the circle that is a cell and just draw a cell without these. It is quite confusing as is. Would write RNA/DNA in y-axis for MPRA. Would run this figure through people who do not know MRPA/STARR-seq and see if they got it and get input from other published figures also.

We’ve made the following suggested edits to improve clarity: (i) we made the GFP green; (ii) we noted that “promoter” is “minimal promoter” in the legend; (iii) we created a second figure where the analysis outputs (RNA/DNA ratio) are shown in more detail; and (iv) we replaced the transfection figure with a dish of cells as well as a close up of a single cell. We believe the poly-A signal is an important part of the vector design and have therefore opted to leave this in. We also tried adding lines to the barcodes but they were visually distracting and detracted from the simplicity that we are aiming for with the figure.

'Additionally, STARR-seq allows the researcher to use captured, immunoprecipitated, or otherwise selected genomic DNA fragments, as well as randomly fragmented DNA' This is incorrect, as MPRA also allows that. Would instead mention that for both sequences can be either oligosynthesized, PCRed, captured or immunoprecipitated. This also needs to be corrected later when the authors mention 'An alternative approach is to use STARR-seq family methods' as again MPRA can and has used these cloning approaches also.

We’ve tempered the language to make it clear that it is most common to use synthesis-derived material in classic/barcoded MPRAs and genomic-derived material in STARR-seq, but that there is indeed some flexibility.

Also, while the advantages of STARR-seq relative to classic MPRA are mentioned, the converse is not. For example, the multiplicity of barcodes associated with each candidate enhancer in MPRA design shields it from the sequence-specific mRNA stability defects present in STARR-seq. Both techniques have limitations and advantages and this should be accurately represented. As mentioned also below in minor comments, would be good to reference the reader to PMID 33046894 (reference 84) which compared 9 different MPRA and possibly discuss this a bit with the limitations of each method and how comparable they were from this article (standard transfection, ORI STARR-seq and lentiMPRA all correlated well to one another and each had various limitations) sending the reader to this article for more detail.

Thanks for pointing this out, we’ve added text to highlight that there are advantages of MPRAs over STARR-seq, and we point the reader to PMID 33046894 for further reading as suggested.

Line 57, page 4: DNA-seq is also a readout of MPRA, which is necessary for normalizing the RNA-seq output by the transfection efficiency of each tested candidate enhancer. Similarly, in Figure 1B, DNA-seq should be included in the STARR-seq workflow. Would also provide more general text on how MPRA or STARR-seq measurements are done. What is used to determine if a sequence is active. Can also reference tools to do this, such as MPRAflow, MPRAanalyze, STARRPeaker and others. This will be helpful for readers who might be fearful of this technology that the authors are trying to entice, that also the computational tools are available for it.

We agree that the analysis side of MPRAs was not adequately discussed in the first version of our manuscript. We’ve added a new figure (Figure 2) and section (Box 1) to specifically discuss analysis workflows and the pros/cons of different approaches as well as general challenges. In addition, we have made clear that both RNA and DNA are readouts of an MPRA, and that the latter is used to normalize the former.

In the 'These cell numbers can be prohibitive in the case of hard-to-transfect' paragraph, another option could also be carrying out MPRA in the organism of interest. MPRA was done in mice, via hydrodynamic tail vein injections or AAV, flies, ciona and could potentially be developed in other organisms. Would add text to mention this as another approach.

Added.

There are several mentions of using MPRA to study phenotypic evolution and divergence. While MPRA is described succinctly, a description of how data from an MPRA-style experiment can then be linked to target genes, traits and phenotypes is lacking.

We agree this is important and have included information on this type of analysis in our new text about statistical analyses.

Figure 3 feels more like a supplemental figure. Similar to Figure 2, would maybe have instead for Figure 3 in this section the various recommendations for expanded usage.

We’ve moved Figure 3 to the SI and have added a new figure focused on data analysis since this was a key missing piece noted by several reviewers.

Would remove 'in vitro' in abstract from 'single in vitro experiment' as MPRAs have also been carried out in vivo.

Done.

Would remove 'explicitly' in 'to address explicitly evolutionary'

Done.

'although often within 1 megabase7,10'as some genomes discussed here will be much smaller, to not confuse readers, would mention for what genomes this work determined with 1 megabase.

Good point, modified accordingly.

Line 25 on page 3: the author mentioned that enhancer regulates transcription by recruiting RNA polymerase II (Pol II). Though Pol II can be recruited by some enhancers, it's mostly recruited to promoters near transcription start sites.

Modified.

Line 28 on page 3: change 'Enhancers' to 'Candidate enhancers', as the number of functionally validated enhancers is far less than the number of protein-coding genes.

Done.

Line 55 on page 4: change 'to a unique barcode' to 'unique barcodes'. In most cases, a sequence tested in MPRA is linked to multiple barcodes.

Done.

'This is largely because they are difficult to identify from genomic or epigenomic datasets' I would also add another important hurdle here that enhancers are tissue/cell type specific and also context or temporal specific, i.e. can only be on if due to some perturbation or at a certain developmental time point for example. Due to these they are also hard to identify.

Agreed, modified accordingly.

'lead to a reduction' change to 'leads to a reduction'.

Thanks for the close read. In the text, 'lead' refers to snake-specific mutations in plural, so we've left this one unchanged.

' these changes to the design impact the types of information that can be gained from a given assay (Table 1).' Might want to finish this sentence with some words referencing PMID 33046894 which compared 9 different MPRA techniques to send the reader to read more and understand what method might be good for them.

Agreed, modified accordingly.

'Further, several detailed MPRA protocols are now publicly available75,76' Would also add PMID 32641802 which provides a detailed protocol (reference 38).

Done.

In the section named 'CURRENT APPLICATIONS OF MPRAS', most discussions were focused on MPRA studies in the evolutionary field. Thus, it is more appropriate to name the section 'CURRENT APPLICATIONS OF MPRAS IN EVOLUTION'. Also, since the current applications of MPRAs were just briefly touched up here, it is necessary to mention that this is beyond the scope of this article and refer readers to other more comprehensive reviews of MPRA.

We’ve opted to keep the more general header since the first paragraph does not focus on evolutionary studies. We have added text to say that a thorough review of biomedical applications is outside the current scope.

Line 47 on page 7: the 14k SNPs are found in modern humans but not in archaic hominins and great apes. The author didn't mention great apes.

Edited this sentence to clarify.

Since section 'EXPANDING MPRA USAGE IN ECOLOGY AND EVOLUTION' mostly talked about things that could be done in the future. It would be better to combine this section with 'FUTURE DIRECTIONS AND CONCLUSIONS.

We’ve changed 'FUTURE DIRECTIONS AND CONCLUSIONS’ to 'POTENTIAL FUTURE DIRECTIONS AND CONCLUSIONS’ to address the reviewer’s point . We’ve also modified the first sentence of this section to make it clear that the future directions piece is more speculative than the expanding use section.

In Figure 2 weird to have B below and C next to A. Would move things around.

This sizing of the figure panels doesn’t work to put B next to A, so we’ve opted to leave it as is. We could also switch the order in which panels are called out, but it would no longer match the text.

**Comments from reviewer 3:**

This is a timely and useful review of MPRA technology and its potential use to address evolutionary questions. The authors nicely summarize recent developments in this field, and correctly point out that advances in biomedical applications of MPRAs can just as easily be transferred to evolutionary questions. The review clearly describes MPRA technology, cites major references (the field is much too large to cite comprehensively in a short review), and provides helpful tables to guide readers unfamiliar with the technology.

Thank you for your kind words!

While the authors discuss some of the technical challenges of implementing MPRAs, this review would benefit from a short discussion of the limitations and interpretive challenges of MPRA data. The authors note the importance of cell type, which is a critical limitation when MPRAs are used to test SNPs function. Often the correct cell type is not only unavailable, but also unknown when candidate SNPs and cis-regulatory elements are selected from sequence alone, as in GWAS, cross-species comparisons, human accelerated regions, etc. The choice of basal promoter can determine which elements are active in the assay. If a cis-regulatory element with a potentially important SNP is inactive in the assay, the effect of the SNP cannot be assessed. There is also a question of what counts as a meaningful effect size for a variant in an MPRA. In some cases MPRAs are able to detect small effect sizes, but it is not clear whether those are phenotypically meaningful. On the other hand, given that cellular context is important, it is not clear that SNPs with the largest effects in MPRAs are the most functionally relevant within their genomic and cellular context.

These are all incredibly important points. We’ve added a new section (Box 1) about analysis and interpretation to cover these points and those of the other reviewers.

Related to these issues are questions of what controls to include in MPRAs. For example, when using MPRAs to fine map alleles from scans for positive selection, should the MPRA library include a control set of alleles from neutrally evolving loci in order to estimate the assay's false positive rate?

This is also an important point, we’ve added some text on including proper controls.

MPRAs are a powerful technology, but interpretation of the results is an important issue that should receive more emphasis.

Agreed! As noted in previous responses, we’ve added new text (Box 1) about analysis approaches and interpretation.

**Second round of review**

**Reviewer 2**

The authors have addressed my comments
